# Brachial plexus blockade arising from a combined pectoralis (PECS) 1 and 2 block

**DOI:** 10.1002/anr3.12251

**Published:** 2023-11-05

**Authors:** J. D. Mathers, A. Engum, G. Galleberg

**Affiliations:** ^1^ Department of Anaesthesia Haukeland University Hospital Bergen Norway

**Keywords:** brachial plexus block, local anaesthetic, mastectomy, proprioception

## Abstract

We present a case of inadvertent spread of local anaesthetic from combined pectoralis (PECS) 1 and 2 fascial plane blocks that resulted in an incomplete brachial plexus block. An otherwise healthy 42‐year‐old woman with a body mass index of 23.3 kg.m^−2^ presented for unilateral mastectomy with immediate prosthetic reconstruction for breast cancer. No axillary dissection was performed. Because of service requirements, the blocks were performed at the conclusion of surgery. This may have resulted in greater cranial spread of the local anaesthetic due to surgical dissection along musculature and placement of the breast implant. Following emergence from general anaesthesia, the patient experienced numbness over the ipsilateral medial forearm extending to the little finger. Further examination with a finger‐nose test revealed reduced coordination and joint proprioception of the ipsilateral arm. There was no detectable gross motor weakness. She was reviewed the following day (23 h after the blocks) by which time her symptoms had subsided entirely. We believe that this is the first documented brachial plexus block after injection of local anaesthetic into the pectoralis 1 and 2 fascial planes.

## Introduction

Pectoralis (PECS) blocks are an established regional anaesthesia technique for patients undergoing breast surgery, used to reduce postoperative pain and opioid requirements [1]. Two variants are described in the literature. The PECS 1 block involves injection of local anaesthetic between the pectoralis major and minor muscles [2]. The PECS 2 block (sometimes called modified PECS 1) involves injection of local anaesthetic between pectoralis minor and serratus anterior muscles [3]. Both are dependent on volume for effect, and a volume of 10 to 20 ml of local anaesthetic is typically used. Evidence from a cadaver study simulating an infraclavicular block with 20–30 ml of dye identified fascial layers in the infraclavicular region that can impede the spread of injectate [4]. Here, we report the case of a patient who received combined PECS 1 and 2 blocks at the end of surgery after a mastectomy with placement of a submuscular breast implant. It is possible that much of the fascial anatomy may have been disturbed by the surgery, which may have led to the extended spread of local anaesthetic to the brachial plexus.

## Report

A 42‐year‐old woman presented for mastectomy and immediate submuscular implant reconstruction as part of treatment for ductal carcinoma in situ. There was no axillary dissection. She had no other significant medical history, no allergies, and had undergone uncomplicated anaesthesia before. She was 163 cm in height, weighed 63 kg (BMI 23.3 kg.m^−2^) and took citalopram for mild depressive symptoms.

General anaesthesia was induced and maintained with total intravenous anaesthesia with target‐controlled infusions of propofol and remifentanil. The patient's lungs were ventilated via a supraglottic airway in an air and oxygen mixture. Combined PECS 1 and 2 blocks were planned immediately after induction of anaesthesia but because of a lack of available personnel, it was necessary to perform the blocks following completion of the surgical procedure. She underwent a subcutaneous mastectomy with implantation of a 180 ml breast implant under the pectoralis major muscle. Her surgical course was unremarkable and at the end of surgery whilst under general anaesthesia, unilateral PECS 1 and 2 blocks were performed using an ultrasound‐guided aseptic technique. In total, 40 ml of 0.375% ropivacaine was used, with 15 ml injected beneath pectoralis major (PECS 1 block) and 25 ml beneath pectoralis minor (PECS 2 block) at the level of the third rib. The only notable finding during the procedure was a hyperechoic artefact under pectoralis major muscle suggestive of air from surgical dissection. There was no surgical infiltration of local anaesthetic.

The patient had an uneventful emergence from anaesthesia with minimal postoperative pain, indicating effective PECS 1 and PECS 2 blocks. Pre‐operatively she was given 1.5 g of paracetamol, 12 mg of dexamethasone and 10 mg of sustained‐release oxycodone orally. She received 100 mcg of fentanyl intravenously immediately before surgical incision. The total operating time was two and a half hours. A further 50 mcg of intravenous fentanyl was given on arrival to the post anaesthesia care unit (PACU) and 5 mg of oxycodone was administered orally. Approximately 45 min after the end of surgery, the anaesthetic team was called to PACU because the patient was complaining of numbness in her little and ring fingers on the operative side. Further examination revealed anaesthesia of the ulnar aspect of the forearm on the operative side in addition to numbness over her little finger and lateral aspect of her ring finger. This pattern was consistent with the blockade of both the ulnar nerve and the medial cutaneous nerve of the forearm. The patient also demonstrated significantly reduced joint position sense and decreased coordination of the whole arm. Motor function was preserved. We suspect that the inadvertent spread of local anaesthetic to the brachial plexus with medial cord blockade had occurred, rather than direct ulnar nerve injury. We came to this conclusion as there was a combination of proprioceptive loss and paraesthesia in the ulnar nerve territory and blockade of the medial cutaneous nerve of the forearm, which arises independently from the brachial plexus. The anaesthesia team reviewed the patient the following day (approximately 20 h after the PECS blocks). By this time, her sensory and proprioceptive symptoms had resolved. The patient was reassured and discharged home later that day. A follow‐up telephone consultation 2 days later showed no return of her symptoms and she remained pain free.

## Discussion

Inadvertent spread of local anaesthetic is a known complication of many regional anaesthetic techniques. For example, avoiding phrenic nerve palsy associated with interscalene blocks is the rationale for the development of the lower‐dosed interscalene block and the superior trunk block [5, 6]. Both the PECS 1 and PECS 2 blocks are fascial plane blocks and although the targets of anaesthesia are the pectoral nerves and the lateral cutaneous branches of the intercostal nerves, there is the possibility of blockade of the thoracodorsal nerve and long thoracic nerve due to their close anatomical proximity. However, neither the thoracodorsal nor the long thoracic nerve innervate the arm itself nor are they involved in joint proprioception. A systematic review of 637 patients receiving both serratus anterior plane and pectoralis blocks identified no reports of brachial plexus involvement [7]. However, Kulkarni et al report brachial plexus blockade after attempted pectoralis blocks with 10 ml of ropivacaine 0.2% to PECS 1 and 20 ml ropivacaine 0.2% to PECS 2 [8], for a patient listed for a mastectomy. The blocks were performed before induction of anaesthesia and the patient complained of motor weakness and paraesthesia of the ipsilateral arm before induction of anaesthesia. Dissection during surgery identified fluid pooled in the axillary fossa and the patient required higher doses of intra‐operative opioid analgesia than was expected. This was interpreted by the authors as failure of the PECS blocks. In addition, the brachial plexus blockade persisted for 5 days, which is significantly longer than expected for a PECS block carried out with 0.2% ropivacaine [8].

Traditionally the PECS 1, PECS 2 and serratus anterior plane blocks are performed before the start of surgery. The advantages of this include that there is less anatomical distortion from surgery and the analgesic effect of the block is established before the surgical stimulus. Whilst placing a PECS block postoperatively is not directly contraindicated, our case highlights the potential for inadvertent brachial plexus block, possibly due to enhanced proximal spread of local anaesthesia. We postulate that the dissection along anatomical planes during the mastectomy surgery with immediate reconstruction allowed for additional proximal spread of local anaesthetic in our case. We would therefore advise that pectoralis blocks should be performed before starting surgery to avoid inadvertent brachial plexus blockade. Additionally, as part of informed consent, it may be advisable to inform patients of the potential for blockade of the brachial plexus despite this being a temporary and relatively rare complication.
